# Who will go the extra mile? Selecting organizational citizens with a personality-based structured job interview

**DOI:** 10.1007/s10869-020-09716-1

**Published:** 2020-10-07

**Authors:** Anna Luca Heimann, Pia V. Ingold, Maike E. Debus, Martin Kleinmann

**Affiliations:** 1 Department of Psychology, University of Zurich, Binzmuehlestrasse 14/Box 12, 8050 Zurich, Switzerland; 2 School of Business, Economics, and Society, Friedrich-Alexander University Erlangen-Nürnberg, Erlangen, Germany

**Keywords:** Organizational citizenship behavior, Personnel selection, Structured interviews, Personality traits

## Abstract

Employees’ organizational citizenship behaviors (OCB) are important drivers of organizational effectiveness. Yet, there exist no established tools for selecting employees with a propensity to engage in OCB. Given that personality traits describe typical behavioral tendencies and are established OCB predictors, we propose that personality assessment is a useful approach for selecting employees who are likely to exhibit OCB. To test this proposition, we developed a structured job interview measuring the Big Five traits and then compared this interview to a personality self-report measure to determine which method of personality assessment works best for selecting organizational citizens. Employees (*N* = 223) from various occupations participated in the structured job interview and completed the personality self-report in a simulated selection setting. We then obtained supervisor ratings of employees’ OCB. Results supported the assumption that structured job interviews can be specifically designed to assess the Big Five personality traits and, most importantly, to predict OCB. Interview ratings of specific personality traits differentially predicted different types of OCB (i.e., OCB-compliance, OCB-helping, and OCB-initiative) and explained incremental variance in OCB over and above personality self-reports and verbal cognitive ability. Taken together, these findings expand our knowledge about dispositional predictors of OCBs, personality assessment in selection, and the design of job interviews.

Organizations can benefit greatly from employees who engage in organizational citizenship behaviors (OCB; Koys, 2001; Podsakoff, Whiting, Podsakoff & Blume, 2009). By definition, OCB refers to behaviors that contribute to the social and psychological context at work—such as upholding the rules, helping others, and taking initiative in advancing the organization (Organ, 1997; Organ, Podsakoff & MacKenzie, 2006). Underlining its importance in the organizational context, OCB has been termed the “social lubricant” that keeps an organization running (Smith, Organ & Near, 1983) or the “catalyst” that sparks task performance (Borman & Motowidlo, 1997). As such, OCB has generated an enormous research *interest*: A simple PsycINFO search shows that there were over 3000 publications revolving around OCB and related constructs. Given OCB’s relevance, researchers concluded that “it would appear worthwhile for organizations to select employees who demonstrate a predisposition to exhibit OCB at work” (Organ, Podsakoff & Podsakoff, 2011, p. 294). Yet, despite repeated calls to explicitly consider OCB as a criterion in personnel selection, research on *how* to predict OCB in selection settings has been surprisingly scarce (Borman & Motowidlo, 1997; Organ et al., 2011; Werner, 2000).

In the present study, we aim to answer the aforementioned call by exploring a new approach for selecting organizational citizens: a personality-based structured job interview. We predict (a) that structured job interview questions will be useful tools for assessing manifestations of the Big Five personality traits, (b) that employees who are high in specific personality traits (i.e., Conscientiousness, Agreeableness, and Intellect/Openness) will be likely to engage in specific OCBs (i.e., OCB-compliance, OCB-helping, and OCB-initiative), (c) that personality-based interview ratings will explain variance in OCBs above and beyond other measures such as a personality self-report and a test of verbal cognitive ability, and (d) that personality-based interview ratings will assess specific manifestations of personality and therefore will mediate the relationship between underlying personality traits and OCBs.

We focus on the assessment of personality traits because they tend to be considered major predictors of motivational (i.e., “will-do”) performance criteria like OCB (Cortina and Luchman, 2013; Motowidlo, Borman & Schmit, 1997). As compared to other OCB predictors (i.e., job attitudes; Hoffman, Blair, Meriac & Woehr, 2007), personality traits may be relatively stable within a given domain (e.g., in the context of work; Judge, Simon, Hurst & Kelley, 2014; Woods & Hampson, 2010). Hence, employees are likely to “bring” their personality with them when they start a new job (see also Sackett, Lievens, Van Iddekinge & Kuncel, 2017), and this can make it viable to assess personality in order to identify those employees who possess a predisposition to engage in OCB.

Moreover, the structured job interview seems a feasible method for assessing personality traits in the context of selection (Levashina, Hartwell, Morgeson and Campion, 2014). One reason may be that structured interviews are part of many selection procedures (e.g., Jackson, Dewberry, Gallagher & Close, 2018; König, Klehe, Berchtold & Kleinmann, 2010). They are standardized (e.g., all interviewees answer the same questions; Campion, Palmer & Campion, 1997), they are perceived more favorably by applicants than traditional personality measures (i.e., self-report questionnaires; Anderson, Salgado & Hülsheger, 2010), and they have consistently shown to predict job performance (Huffcutt, Culbertson & Weyhrauch, 2014; McDaniel, Whetzel, Schmidt & Maurer, 1994). In this regard, it is important to note that the content of structured interviews needs to be *job-related* in order for the interview to predict job performance (Campion et al., 1997; McDaniel et al., 1994). Following this rationale, interview questions should refer to the context of work if they aim to assess personality traits to predict OCB. This is in line with Levashina et al. (2014) who post that in order to assess personality with structured interviews, interview questions should be “designed to measure the specific job-related behaviors that are presumed to underlie a particular personality trait” (p. 265).

By combining research on OCB, personality assessment, and job interviews, this study contributes to the literature in several ways. First, we provide a novel examination as to whether certain personality traits within the realm of the Big Five (Goldberg, 1990; Hofstee, de Raad & Goldberg, 1992) differentially predict different types of OCBs. Hence, this study aims at expanding our knowledge about which personality traits are most relevant for predicting specific citizenship behaviors. Second, this study is among the first to examine relationships between all Big Five traits and different types of OCB in the context of personnel selection (see for example Wang & Bowling, 2016). Even though the selection context affects how participants report their personality (Birkeland, Manson, Kisamore, Brannick & Smith, 2006; Schmit & Ryan, 1993), previous research on personality and OCB has normally been conducted in settings that do not reflect or simulate selection situations (e.g., Kluemper, DeGroot & Choi, 2013; Wang & Bowling, 2016). Third, this study introduces a job interview designed to assess manifestations of the Big Five traits and, thus, informs the debate on which methods (other than traditional self-report measures) can be used to assess personality in the context of selection (e.g., Morgeson et al., 2007; Ones, Dilchert, Viswesvaran & Judge, 2007; Rothstein & Goffin, 2006; Salgado, 2016). Fourth, we aim to deepen our conceptual understanding as to why interview ratings are adequate OCB predictors. For this purpose, we test the assumption that interview ratings capture specific expressions of personality traits which link underlying personality traits to citizenship behaviors.

## OCB as a criterion in personnel selection

Previous research has placed attention on OCB as a potential criterion in personnel selection (Borman & Motowidlo, 1993; Organ et al., 2011; Werner, 2000) because OCB is typically regarded as an integral part of the larger job performance domain (Hoffman et al., 2007; Koopmans et al., 2011; Viswesvaran & Ones, 2000). In line with this, supervisors and decision-makers tend to take OCB into account when evaluating employees’ job performance (Podsakoff et al., 2009; Podsakoff, Whiting, Podsakoff & Mishra, 2011), and research has illustrated that OCBs are related to a number of important organizational outcomes such as higher productivity and lower turnover rates (Koys, 2001; Podsakoff et al., 2009).

Research has further identified different types of citizenship behaviors that are directed at benefiting the context of work in different ways (e.g., Organ et al., 2006). In line with previous research on OCB in the context of selection (Allen, Facteau & Facteau, 2004; Podsakoff et al., 2011), the present study focuses on three forms of OCB: *compliance*, *helping*, and *initiative*. OCB-compliance and OCB-helping have received considerable attention because they constitute the first OCB dimensions identified in the literature (Organ et al., 2006; Smith et al., 1983). OCB-compliance can be characterized by following the rules of the organization and by showing low levels of absenteeism (Smith et al., 1983). Hence, OCB-compliance is often understood as a form of OCB directed at the organization (i.e., OCB-O; Williams and Anderson, 1991). OCB-helping includes prosocial behaviors such as preventing conflicts among co-workers, helping other co-workers with their tasks when needed, and cheering co-workers up (Organ et al., 2006). Thus, OCB-helping is a form of OCB directed at other individuals (i.e., OCB-I; Williams & Anderson, 1991). More recently, change-oriented forms of OCB, such as OCB-initiative, have received considerable attention in research on job performance (Chiaburu, Oh, Berry, Li & Gardner, 2011; Marinova, Peng, Lorinkova, Van Dyne & Chiaburu, 2015). OCB-initiative refers to employees’ active and constructive involvement in the governance of the organization and has also been labeled as civic virtue (Graham, 1986; Konovsky & Organ, 1996; Organ, 1988; Organ et al., 2006). It can encompass behaviors such as keeping oneself informed about what is going on in the organization and making innovative suggestions for change.

## Personality traits as predictor constructs

Conceptually, personality traits, such as the Big Five (Extraversion, Emotional Stability, Conscientiousness, Agreeableness, and Intellect/Openness) describe how individuals typically think, feel, and behave (Goldberg, 1990, 1992). According to theories of job performance, personality traits determine the knowledge, skills, and habits individuals acquire, and thereby shape how individuals behave at work (Bergman, Donovan, Drasgow, Overton & Henning, 2008; Dudley & Cortina, 2008; Motowidlo et al., 1997). In support of this, meta-analyses provide some evidence that the Big Five traits relate to different citizenship behaviors (Borman, Penner, Allen & Motowidlo, 2001; Chiaburu et al., 2011; Dalal, 2005; Hurtz & Donovan, 2000; Ilies, Fulmer, Spitzmuller & Johnson, 2009; LePine, Erez & Johnson, 2002; Marinova et al., 2015; Organ and Ryan, 1995).

Although there is a consistent pattern of findings that encourages the use of personality traits for predicting OCB, it is important to note two limitations: First, extant research on dispositional OCB predictors has relied to a large degree on traditional self-report measures for assessing personality (e.g., Organ et al., 2011). This might be problematic because personality self-report measures—in selection contexts—have been criticized for being more vulnerable to intentional response distortion than other selection instruments (e.g., structured interviews; Van Iddekinge, Raymark & Roth, 2005). Second, and relatedly, there are very few studies that have examined the relationships between personality traits and OCB within an actual or simulated selection context. Two studies using data from actual selection settings (Anglim, Lievens, Everton, Grant & Marty, 2018; Hogan, Rybicki, Motowidlo & Borman, 1998) and one study with data from a simulated selection setting (Blickle, Momm, Schneider, Gansen & Kramer, 2009) found that different personality traits predict OCB. Yet, these studies (a) employed self-reports only for assessing personality traits, (b) did not all differentiate between several types of OCB, and (c) found somewhat inconsistent patterns of results (i.e., Conscientiousness being predictive of OCB in Blickle et al., 2009; versus Adjustment, Prudence, and Ambition being predictive of OCB in Hogan et al., 1998; versus Agreeableness and Extraversion being predictive of OCB in Anglim et al., 2018). Specifically, in the selection context, exploring methods other than self-reports might offer a promising opportunity to improve personality assessment and thereby the pinpoint prediction of different OCBs.

## Structured interviews as predictor methods

Structured interviews are widely established selection methods with features that may improve the assessment of personality predictors in personnel selection (Levashina et al., 2014). Specifically, personality measures have shown to be better at predicting work-related performance criteria when they are contextualized (i.e., designed to match the context of work) and, thereby, provide a clear frame-of-reference for applicants (Shaffer & Postlethwaite, 2012; Wang & Bowling, 2016). Job interviews may be regarded as contextualized measures because the content of interview questions is likely to be associated with the context at work (e.g., Levashina et al., 2014). In addition, interviews feature an open response format (see Raymark & Van Iddekinge, 2013). This open response format places high cognitive demands on applicants and has been found to decrease intentional response distortions in comparison to self-report responses on personality measures (Van Iddekinge et al., 2005).

Going beyond the assessment of personality traits, the structured interview has already been proposed as a potential method to predict OCB (Organ et al., 2011; Werner, 2000). Two studies have examined structured job interviews as predictors of OCB so far, but none of them has used a personality-based interview. Latham and Skarlicki (1995) and Allen et al. (2004) both developed interview questions for assessing OCB and found that their interview ratings partly predicted co-worker ratings of OCB. Despite these first promising findings, some fundamental questions remain to be answered because previous studies have yielded inconsistent results. The former study (Latham & Skarlicki, 1995) found that only some of the developed interview questions predicted OCB (i.e., behavior description interview questions did not predict OCB), and the latter study (Allen et al., 2004) reported that interview ratings were only predictive of some of the examined citizenship behaviors (e.g., their interview ratings did not predict OCB directed at other individuals). In addition, both studies did not examine the prediction of supervisor ratings of OCB, but peer-ratings of OCB. In contrast to this approach, employees’ performance in the selection context is most frequently assessed via supervisor ratings (Woehr and Roch, 2012), and supervisor ratings have shown to be the best single predictors of independent performance criteria when compared to self- and peer-ratings (Atkins & Wood, 2002; Darr & Catano, 2008).

## A personality-based structured job interview for predicting OCB

Addressing the limitations of previous research on personality traits and structured interviews as predictors of OCB, the present study has four consecutive purposes: We aim to establish (a) construct-related validity evidence for a structured job interview assessing the Big Five, (b) criterion-related validity evidence for this interview by carefully linking the Big Five traits to supervisor ratings of specific OCBs, (c) incremental validity evidence of personality-based interview ratings over and above personality self-reports and verbal cognitive ability in predicting OCB, and (d) evidence that interview ratings are more proximal to OCB as compared to traditional personality self-reports because interview questions capture specific manifestations of personality.

First, gathering construct-related validity evidence may be a central prerequisite for testing whether a personality-based job interview is a useful approach for selecting organizational citizens (see also Hamdani, Valcea & Buckley, 2014). This is likely to be relevant given that research on assessing personality traits with job interview questions has been scarce: Although interview questions often assess personality-related interview dimensions among many others (e.g., drive, decisiveness, sense of duty, and likability; Huffcutt, Conway, et al., 2001a), these interview dimensions are oftentimes not aligned with established personality frameworks such as the Big Five. In line with this, previous studies examining personality saturation in structured interviews found low correspondence between traditional job interviews and personality self-reports with uncorrected meta-analytic estimates ranging from .01 to .17 (Roth, Van Iddekinge, Huffcutt, Eidson Jr. & Schmit, 2005; Salgado & Moscoso, 2002).

In their review, Levashina et al. (2014) summarized 18 research articles on personality and structured interviews. They identified one study that explicitly developed structured interview questions for assessing personality traits (Van Iddekinge et al., 2005). Although this study only focused on specific facets of three selected traits (i.e., Vulnerability, Altruism, and Self-Discipline) and did not collect criterion data, the findings support the proposition that structured interview questions have the potential to measure established personality constructs (Van Iddekinge et al., 2005). In line with these findings, Levashina et al. (2014) conclude that “research should further examine personality-based structured interviews as an alternative method of applicant personality self-assessment” (p. 264).

When assessing the Big Five personality traits with a structured job interview, we argue that a patterned behavior description interview (see Janz, 1982) is a particularly useful interview format: Behavior description interview questions are thought to primarily capture interviewees’ job experience and personality (Levashina et al., 2014). In this interview type, applicants report their own past behavior in previously encountered situations (Janz, 1982). Given that personality traits are considered to manifest in behaviors (e.g., Tett & Burnett, 2003), evaluating interviewees’ past behaviors in actually experienced situations would allow for insight into interviewees’ personality. Accordingly, we hypothesize that the Big Five personality traits can be measured as dimensions in a behavior description interview. To test this assumption, we posit the following hypothesis:

- *Hypothesis 1*: A factor model specifying the Big Five personality traits as distinct and correlated latent factors will best represent the data structure of the developed interview.

Second, and regarding criterion-related validity, it is conceivable that the Big Five personality traits, being distinct constructs, differentially predict different forms of OCB. In line with this, we test the interviews’ criterion-related validity by establishing differential hypotheses for the prediction of OCB-compliance, OCB-helping, and OCB-initiative, respectively. Thereby, we follow Borman and Motowidlo (1997) who highlight that “evidence toward establishing empirical links between personality constructs and relatively specific criterion constructs contributes importantly to the science of personnel selection” (p. 108).

Specifically, we propose that interview ratings of Conscientiousness are particularly predictive of OCB-compliance—for the following reasons: Individuals scoring high on Conscientiousness tend to feel responsible and act reliably; they are industrious and hard-working (Goldberg, 1990; Hofstee et al., 1992). As they are likely to feel a sense of duty towards their work, conscientious employees may be more likely to engage in behaviors that maintain a productive work environment such as OCB-compliance (see also Chiaburu et al., 2011; Ilies et al., 2009). We conclude that Conscientiousness will be the strongest personality predictor of OCB-compliance, thus arriving at the following prediction:

- *Hypothesis 2*: Conscientiousness assessed in the structured job interview will predict OCB-compliance as rated by supervisors over and above the other Big Five traits.

Furthermore, we hypothesize that interview ratings of Agreeableness are particularly predictive of OCB-helping. Individuals scoring high on Agreeableness value social harmony and tend to be sympathetic, understanding, and cooperative (Goldberg, 1990; Hofstee et al., 1992). As they are striving to have positive relationships with others, agreeable employees may tend to engage in OCB-helping which benefits other individuals at work (see also Chiaburu et al., 2011; Ilies et al., 2009). We expect that Agreeableness is the most relevant predictor of OCB-helping and therefore posit:

- *Hypothesis 3*: Agreeableness assessed in the structured job interview will predict OCB-helping as rated by supervisors over and above the other Big Five traits.
  In addition, interview ratings of Intellect/Openness should be particularly predictive of OCB-initiative. This is because individuals scoring high on Intellect/Openness are imaginative, independent, creative, curious, and full of ideas (Goldberg, 1990; Hofstee et al., 1992). As they are open to trying new things and are likely to have new ideas, employees high on Intellect/Openness may often engage in OCB-initiative to enhance and advance their work environment (see also Chiaburu et al., 2011; Marinova et al., 2015). Accordingly, we hypothesize that Intellect/Openness is the strongest predictor of OCB-initiative, and thus, we predict:
- *Hypothesis 4*: Intellect/Openness assessed in the structured job interview will predict OCB-initiative as rated by supervisors over and above the other Big Five traits.

A question of more practical relevance is whether the newly-developed personality-based interview shows incremental validity over and above other measures such as a personality self-report questionnaire and a verbal cognitive ability test. Personality questionnaires have the advantage that they are simple to administer, tend to be cost-efficient, and can be easily contextualized (i.e., adapted to reference to the work setting; Shaffer & Postlethwaite, 2012; Wang & Bowling, 2016). Cognitive ability tests are also relatively simple to administer and part of many selection procedures (e.g., Wee, Newman & Joseph, 2014). Previous research demonstrated that cognitive ability is a predictor of both job interview ratings (Berry, Sackett & Landers, 2007; Roth & Huffcutt, 2013) and OCB (Gonzalez-Mulé, Mount & Oh, 2014). Hence, the criterion-related validity of interview ratings in predicting OCB could potentially be driven by interview ratings capturing cognitive ability. In particular, *verbal* cognitive ability (e.g., having a rich vocabulary, being able to express oneself) seems relevant to interview ratings, given that interviewee performance is thought to be influenced by the verbal content of interview responses and the verbal delivery of these responses (Huffcutt, Van Iddekinge & Roth, 2011). Therefore, investigating whether the interview explains variance in OCB beyond both a personality self-report questionnaire and a verbal cognitive ability test is warranted to determine if the costs of developing and administering a personality-based interview are justified (Barrick, Patton & Haugland, 2000; Macan, 2009; Van Iddekinge et al., 2005).

More specifically, a personality-based structured interview may be more predictive of OCB than a contextualized self-report questionnaire as the interview seems to allow for a more comprehensive assessment of applicants’ personality. This is because the interview method features an open response format and asks for applicants’ cognitions, emotions, and behaviors in specific situations. In line with this, Raymark and Van Iddekinge (2013) pointed out that personality-based interviews are especially rich in information as they “require the applicant to generate detailed responses to job-related scenarios” (p. 428). These detailed responses provide the interviewers with comprehensive information on how the applicant perceives a number of job-related situations and how the applicant chooses to behave in these situations. Hence, we make the following predictions regarding the incremental validity of the personality-based interview:

- *Hypothesis 5a*: Conscientiousness assessed in the structured job interview will explain variance in OCB-compliance as rated by supervisors over and above contextualized self-reports of Conscientiousness and verbal cognitive ability.
- *Hypothesis 5b*: Agreeableness assessed in the structured job interview will explain variance in OCB-helping as rated by supervisors over and above contextualized self-reports of Agreeableness and verbal cognitive ability.
- *Hypothesis 5c*: Intellect/Openness assessed in the structured job interview will explain variance in OCB-initiative as rated by supervisors over and above contextualized self-reports of Intellect/Openness and verbal cognitive ability.

Finally, it is vital to better understand how personality and OCB are linked to each other and *why* the interview method should be a useful approach for identifying organizational citizens. An explanation as to why interview ratings are predictive of OCB is that the interview method can capture situation-specific manifestations of personality that are most proximal to performance-relevant behavior in the workplace such as OCB. While personality self-reports are supposed to assess relatively stable dispositions (e.g., Hough & Furnham, 2003; Sackett et al., 2017; Wrzus & Mehl, 2015), interview ratings focus on how these dispositions reflect on employees’ thoughts, feelings, and behaviors in given situations. These situation-specific manifestations of personality might be good indicators of whether (or to what extent) employees exhibit OCB because it is in these situations that employees decide to engage in behaviors such as following rules, helping others, or trying to change something within the organization. If these assumptions hold true, interview ratings of personality traits (i.e., cognitive, emotional, and behavioral manifestations of personality) should mediate the relationship between self-reported personality traits (i.e., general dispositions) and OCB (i.e., performance-relevant behaviors at the workplace). Accordingly, we posit:

- *Hypothesis 6a*: Interview ratings of Conscientiousness will mediate the relationship between self-reported Conscientiousness and supervisor-rated OCB-compliance.
- *Hypothesis 6b*: Interview ratings of Agreeableness will mediate the relationship between self-reported Agreeableness and supervisor-rated OCB-helping.
- *Hypothesis 6c*: Interview ratings of Intellect/Openness will mediate the relationship between self-reported Intellect/Openness and supervisor-rated OCB-initiative.

## Method

### Sample

#### Interviewees

Interviewees were 223 (91 women, 132 men) individuals who completed a job interview in a simulated selection setting at a European university. We recruited interviewees through the career services departments of vocational training institutions, several universities, social media, and advertisements in local newspapers. Interviewees participated in the simulated selection procedure to prepare themselves for future job applications. In return for their participation, they received comprehensive performance feedback and general advice on job applications. As a precondition, all interviewees had to be employed and were asked to provide the contact details (name and e-mail address) of their direct supervisor upon signing up for the study so that we could collect supervisor ratings of interviewees’ OCB. Mean age of interviewees was 30.56 (*SD* = 7.32) years. Most interviewees (82%) held an academic degree, and more than half of interviewees (63%) had already participated in three or more formal job interviews. They had been working in their current job for 2.57 (*SD* = 2.22) years on average. Interviewees were asked to categorize their current jobs. In general, we found that interviewees held a great variety of different jobs. Almost one third of interviewees (30%) indicated that they had a job as researchers and developers, 12% worked as administrative assistants, 10% worked as project managers, 8% as financial analysts or accountants, 6% worked as instructors or lecturers, 4% worked as IT professionals, 4% as sales persons, 3% as human resources managers, 3% as marketing professionals, 3% worked as supply chain managers, 2% had a job as technical staff, 2% worked as customer service representatives, 2% as public relations professionals, 2% as health service professionals, 1% as executive managers, 1% as quality assurance managers, 1% as media professionals, and 5% did not indicate any of these categories.

#### Supervisors

Supervisors received a link to an online questionnaire that included demographic questions and questions on interviewees’ OCB. Supervisors’ response rate was 90% (i.e., the questionnaire was returned for 200 out of the 223 interviewees). In total, 198 supervisors (56 women, 142 men) completed the questionnaire; of these, 196 supervisors rated one interviewee and two supervisors rated two interviewees. Supervisors’ mean age was 44.27 (*SD* = 9.88) years. Most of them (83%) had been working together with the rated interviewee for more than 1 year. They reported that they could evaluate interviewees’ behavior on the job well on a scale from 1 = *badly* to 5 = *well*, with a mean score of 4.61 (*SD* = 0.62) and a mode score of 5. We did not exclude any supervisor from the sample because none of them indicated that they were not able to evaluate interviewees. Supervisors did not receive any information about their employees’ performance in the simulated selection procedure (i.e., supervisors did not have access to interview ratings or personality scores), and supervisor ratings were confidential (i.e., interviewees did not have access to their supervisor’s rating).

#### Interviewers

Interviewers were 78 (61 women, 17 men) advanced psychology students who were on average 28.04 (*SD* = 7.91) years old and had studied psychology as a major for 6.68 (*SD* = 2.28) semesters on average. Prior to participating in this study, interviewers had completed a 1-day frame-of-reference interviewer training (Roch, Woehr, Mishra & Kieszczynska, 2012; Woehr & Huffcutt, 1994). During this training, interviewers were informed about the position they were interviewing for (i.e., they received a job ad), learned about the interview dimensions (here: the Big Five personality traits), and were given the opportunity to practice administering and rating the interview questions in small groups. They were provided with an interview guide which had instructions on how to start and end the interview in a standardized manner and on how to behave during the interview (e.g., they could repeat each interview question once but probing was not allowed in the interview). Interviewers had no access to interviewees’ self-reports and ratings from interviewees’ supervisors, and they were blind to the hypotheses of this study.

### Procedure

We used a simulated selection setting similar to those that have been successfully employed in previous studies (e.g., Barrick et al., 2000; Swider, Barrick & Harris, 2016; Van Iddekinge et al., 2005; Van Iddekinge, Raymark, Roth & Payne, 2006). We opted to use a simulation because we wanted to examine the interview’s validity in a controlled setting where interviewers do not have previous knowledge about interviewees that may influence perceptions of personality (e.g., their résumé or results from previous tests) and where employed interviewees would allow us to collect OCB ratings from their supervisors. Interviewees were explicitly instructed to behave as if they were applying for a management trainee position. We chose this type of position because it is typically open to individuals from different professional and educational backgrounds. Before starting with the simulated selection procedure, interviewees were given a job ad for this position with a short description of the company, the position, and the skills needed for this position. The same job ad had previously been given to interviewers. The job ad was similar to those described in previous studies using a simulated selection setting (e.g., Ingold, Kleinmann, König & Melchers, 2016).

Interviewees completed a personality-based job interview and a contextualized personality self-report within the simulated selection setting. Selection instruments were presented in randomized order so that half of the interviewees filled in the contextualized personality self-report first, whereas the other half completed the personality-based interview first. The personality-based interview contained 15 interview questions (i.e., three questions per trait) and took 30 min in total. It was possible to conduct the interview within this time frame because the interview was highly structured to prevent non-formalized interactions between interviewers and interviewees. Specifically, interviewers were instructed to closely adhere to the interview guide *not* allowing for (a) paraphrasing or explaining interview questions, (b) reading out interview questions to interviewees more than once, or (c) asking any follow-up questions (i.e., probing). In addition, interviewees were instructed to keep their responses short.

A panel of two interviewers administered each interview. Interviewer pairs were not constant but changed across interviewees to minimize interviewer effects and to assure that interviewers were randomly assigned to interviewees. Interviewers took notes on interviewees’ responses to each interview question and then individually rated the responses. At the end of the simulation, interviewers had time to compare and to discuss their individual ratings for each interview question if their ratings diverged. At the same time, interviewees responded to questions on the perceived authenticity of the simulated selection procedure. Afterwards, interviewees received extensive feedback on their interview performance in order to prepare them for future job applications.

### Measures

#### Personality-based job interview

We developed a behavior description interview (based on Janz, 1982) to assess the Big Five personality traits. The interview assessed specific behaviors at work as indicators of the Big Five personality traits. Interview development proceeded in five steps. First, we collected specific behaviors that (a) were characteristic of the Big Five personality traits and (b) could be observed in the context of work. To collect these behaviors, we considered two established personality questionnaires: the 50-item questionnaire from the International Personality Item Pool (IPIP; Goldberg, 1992) and the NEO Five Factor Inventory (NEO-FFI; Costa & McCrae, 1989; McCrae & Costa, 2004).

Second, the first author of this study developed a pool of 60 interview items describing situations at work in which the previously collected personality-related behaviors can be observed. Co-authors of this study then carefully revised this pool of interview items several times by adapting them to meet the following criteria: (a) interview items had to refer to situations that every interviewee had already experienced at work regardless of their specific jobs, (b) interview items had to refer to situations that elicit behaviors which can be clearly assigned to one personality trait, (c) interview items had to refer to situations that allow for variability in the responses of interviewees, and (d) the structure of interview items (e.g., wordings, length of each item) needed to be similar for all interview items. This process led to a set of 20 interview items.

Third, 5-point rating scales with behavioral anchors for each of the 20 interview items were developed. The first author constructed behavioral anchors that were typical of low, average, and high characteristics of the respective personality trait based on items from personality questionnaires, namely the IPIP (Goldberg, 1992) and the NEO-FFI (McCrae & Costa, 2004), and adapted the behavioral anchors to the context of the interview items. Co-authors of this study then independently reviewed the ratings scales and provided feedback on how to make them applicable for a majority of jobs.

Fourth, to ensure the content validity of interview questions, five subject matter experts (i.e., I-O psychologists specialized in personnel selection; none of whom were co-authors of the present study) provided evaluations on the developed behavior description interview questions consisting of 20 interview items and their respective rating scales. For every interview question, the experts rated how accurately the respective question tapped into each personality trait. In addition, experts indicated if they expected variability in the responses of different interviewees to the interview questions. Furthermore, the experts provided written feedback on the relevance and suitability of the behavioral anchors (i.e., evaluating questions such as “Are behavioral anchors likely to be observable in different kind of jobs?”, “Do behavioral anchors fit with the respective interview item?”). Finally, on the basis of these evaluations, the authors of the present study (a) discarded interview questions that had been evaluated as less effective for assessing the intended personality trait and (b) revised interview items and behavioral anchors according to the feedback provided by subject matter experts.

The final personality interview consisted of 15 behavior description interview questions. Each interview question was designed to measure only one personality trait, and each personality trait was measured with three interview questions. An example interview question for each personality trait is presented in Appendices 1 to 5.

Two interviewers rated interviewees’ responses to each interview question on a 5-point scale ranging from 1 *= not characteristic* to 5 = *highly characteristic* regarding the respective personality trait. After having completed all interviews together, interviewers discussed their individual ratings if these ratings were discrepant by two points or more. Interviewers did not have to agree on the same final rating but were allowed to make final changes to their ratings. To determine interviewers’ interrater reliability, we calculated a one-way random effects ICC for every interview question. Across the 15 interview questions, the ICC for the interviewer panel was .78 and the mean correlation between interviewers’ ratings of each interview question was $\overline{r}$ = .65. Thus, interrater reliability in this study was comparable to the personality-based interview from Van Iddekinge et al. (2005) with a mean ICC of .74 and a mean correlation between interviewers’ ratings of $\overline{r}$ = .60. We then averaged ratings across the two interviewers.

#### Contextualized personality self-report

Interviewees completed the 50-item sample questionnaire from the International Personality Item Pool (IPIP; Goldberg, 1992) which has previously been used by Lievens, De Corte & Schollaert (2008) to obtain contextualized personality self-reports. Similar to previous studies, all items were adapted to the context of work by adding the tag “at work” (e.g., Bowling & Burns, 2010; Wang & Bowling, 2016). Interviewees indicated how accurate each item described themselves on a 5-point scale ranging from 1 *= very inaccurate* to 5 *= very accurate*. Each Big Five personality trait was measured with 10 items. Example items are “At work, I make friends easily” (Extraversion), “At work, I sympathize with others’ feelings” (Agreeableness), “At work, I pay attention to details” (Conscientiousness), “At work, I get stressed out easily” (reverse-coded, Emotional Stability), and “At work, I am full of ideas” (Intellect/Openness). In this study, internal consistencies ranged from *α* = .75 (Conscientiousness) to *α* = .85 (Emotional Stability) and were similar to the internal consistencies reported by Lievens et al. (2008) which ranged from *α* = .76 (Intellect/Openness) to *α* = .89 (Emotional Stability).

#### Verbal cognitive ability

To measure interviewees’ verbal cognitive ability, we used the verbal reasoning module of the IST 2000 (Amthauer, Brocke, Liepmann & Beauducel, 1999), a comprehensive and established cognitive ability test that has previously been used in different fields of psychological research (e.g., Freudenthaler & Neubauer, 2005; Hülsheger, Maier & Stumpp, 2007; Jansen, Lievens & Kleinmann, 2011; Mortensen et al., 2014). The verbal reasoning module comprises three 20-item subtests including tasks such as completing sentences, understanding analogies, and finding similarities. In this study, the internal consistency for the verbal reasoning module was *α* = .81, which was similar to the internal consistency reported by the test developers being *α* = .88 (Amthauer et al., 1999). Previous research reported correlations of this verbal cognitive ability measure with career success to be *r* = .35 and with educational success to be *r* = .43 (Steinmayr & Amelang, 2006).

#### Supervisor ratings of OCB

We measured OCB-compliance, OCB-helping, and OCB-initiative with three subscales from a validated OCB questionnaire from Staufenbiel and Hartz (2000), which is based on the OCB scales from Niehoff and Moorman (1993). All items were rated on a 7-point scale ranging from 1 *= not at all* to 7 *= absolutely*. OCB-compliance was measured with the 5-item subscale labeled *generalized compliance* which has been used in several previous studies (e.g., Debus, Greulich, König & Kleinmann, 2019; Strobel, Tumasjan, Spörrle & Welpe, 2013; Zettler and Solga, 2013). An example items is “This employee follows rules and instructions with great accuracy”. The internal consistency of the scale was *α* = .79. OCB-helping was measured with the 5-item subscale labeled *altruism* (see also Binnewies, Sonnentag & Mojza, 2009; Krumm, Grube & Hertel, 2013; Lehmann-Willenbrock, Grohmann & Kauffeld, 2013). An example item is “This employee helps co-workers if they are overloaded with work”. The internal consistency of the scale was *α* = .84. OCB-initiative was measured with the 5-item subscale labeled *individual initiative* which has been used in previous studies (e.g., Lehmann-Willenbrock et al., 2013; Sackmann, Eggenhofer-Rehart & Friesl, 2009). An example item is “This employee makes innovative suggestions to improve the quality of our work”. The internal consistency of the scale was *α* = .85. Taken together, the internal consistencies of the three scales in the present study were similar to the internal consistencies reported by the authors of the scales, being *α* = .76, *α* = .87, and *α* = .87, respectively (Staufenbiel & Hartz, 2000). A principal factor analysis of all 15 items further supported a three-factor solution that accounted for 51% of the variance in the present sample (average loading on the designated factor was .62). The scale developers reported correlations of these three OCB scales with job satisfaction ranging from *r* = .32 to *r* = .54 and with task-based performance ranging from *r* = .59 to *r* = .63 (Staufenbiel & Hartz, 2000).

#### Further measures

At the end of the simulation, we asked interviewees control questions to check for the perceived authenticity of the selection setting. Interviewees answered the following items on a 6-point scale ranging from 1 *= strongly disagree* to 5 *= strongly agree*: “During the simulation, I behaved as if I would have behaved in an actual selection setting”, “I perceived the selection simulation as realistic”, and “It was easy to adapt to the role of an applicant”.

## Results

We first examined whether interviewees had perceived the simulated selection setting as authentic. Interviewees reported that they behaved as if they were in an actual selection process (*M* = 3.93, *SD* = 0.82, with a mode score of 4 on a scale from 1 *= strongly disagree* to 5 *= strongly agree*), that they perceived the selection simulation as realistic (*M* = 3.59, *SD* = 0.87, with a mode score of 4 on a scale from 1 *= strongly disagree* to 5 *= strongly agree*), and that they could easily adapt to the role of an applicant (*M* = 3.57, *SD* = 0.96, with a mode score of 4 on a scale from 1 *= strongly disagree* to 5 *= strongly agree*). We did not exclude any interviewees from the sample because no interviewee strongly disagreed with all items. Table 1 presents means, standard deviations, and intercorrelations among all study variables.

**Table 1:** Means, standard deviations, and intercorrelations of study variables

Variable		*M*	*SD*	1	2	3	4	5	6	7	8	9	10	11	12	13	14
**1**	Verbal cognitive ability	**38.55**	**7.53**														
Behavior description interview																	
2	Extraversion	3.79	0.58	.05													
3	Agreeableness	3.80	0.55	.09	.31^**^												
4	Conscientiousness	3.96	0.49	− .02	.20^**^	.22^**^											
5	Emotional Stability	3.86	0.51	.09	.32^**^	.25^**^	.22^**^										
6	Intellect/Openness	3.94	0.60	.22^**^	.37^**^	.35^**^	.31^**^	.25^**^									
Contextualized self-reports																	
7	Extraversion	3.68	0.50	.08	.45^**^	.22^**^	.19^**^	.18^**^	.22^**^								
8	Agreeableness	3.88	0.42	.11	.20^**^	.41^**^	.18^**^	.14^*^	.23^**^	.32^**^							
9	Conscientiousness	4.13	0.42	− .02	.09	.01	.30^**^	.04	.08	.04	.23^**^						
10	Emotional Stability	3.91	0.52	.09	.12	.05	.15^*^	.18^**^	.09	.25^**^	.17^*^	.35^**^					
11	Intellect/Openness	3.89	0.47	.32^**^	.25^**^	.06	.02	.13^*^	.39^**^	.43^**^	.27^**^	.15^*^	.14^*^				
Supervisor ratings^a^																	
12	OCB-compliance	5.64	1.01	.03	.04	.14	.22^**^	.09	.01	− .08	.10	.19^**^	.16^*^	− .03			
13	OCB-helping	5.39	0.98	.07	.10	.23^**^	.21^**^	.15^*^	.19^**^	.12	.20^**^	− .05	.08	.09	.53^**^		
14	OCB-initiative	5.58	0.97	.18^**^	.16^*^	.23^**^	.11	.18^*^	.30^**^	.10	.13	.03	.10	.22^**^	.52^**^	.69^**^	

*Note*: *N* = 223

^a^*N =* 200

^*^p < .05; ^**^p < .01, two-tailed

### Assessing personality: Construct-related validity of interview ratings

Hypothesis 1 posited that a factor model specifying the Big Five as dimension factors would best represent the internal structure of interview ratings. To test this hypothesis, we conducted a set of confirmatory factor analyses (CFAs) determining whether interview ratings reflected the Big Five personality traits. Therefore, we used the *lavaan* package (version 0.5-22) for the R environment (Rosseel, 2012). We applied full information maximum likelihood estimation with robust (i.e., Huber-White) standard errors and a robust chi-square test statistic (see also Maydeu-Olivares, 2017). Every latent Big Five personality trait was measured with three interview questions as indicators.

In support of Hypothesis 1, the hypothesized model specifying the Big Five as distinct but correlated dimension factors showed an acceptable fit with *χ*^2^(80) = 104.01, *χ*^2^/*df* = 1.30, *p* = .037, CFI = .93, TLI = .91, RMSEA = .04, and SRMR = .05, which is comparable to the fit reported for the personality-based interview by Van Iddekinge et al. (2005), *χ*^2^(26) = 33.48, *χ*^2^/*df* = 1.29, CFI = .93, and RMSEA = .06.^1^ In addition, we tested two alternative measurement models. In the first alternative measurement model, all interview questions loaded on one general interview factor (i.e., a model typically found to represent interview ratings; Krajewski, Goffin, McCarthy, Rothstein & Johnston, 2006). However, this model did not show an acceptable fit, *χ*^2^(90) = 155.60, *χ*^2^/*df* = 1.73, *p* < .001, CFI = .81, TLI = .78, RMSEA = .06, and SRMR = .06. The second alternative measurement model specified the Big Five as separate but correlated dimension factors and a general interview factor (which is similar to a common method factor). However, this model did not converge. In sum, CFA results demonstrated that the hypothesized model with the Big Five as distinct but correlated factors (and without an additional method/interview factor) showed the best admissible fit and supported Hypothesis 1 concerning the interviews’ construct-related validity.

In addition, we conducted correlational multitrait-multimethod (MTMM) analyses (Campbell & Fiske, 1959) to compare the structure of interview ratings to findings from previous interview studies. The average correlation between the same traits assessed by different methods (i.e., by different interview questions) was .26 (monotrait-heteromethod correlation; convergent validity), and the average correlation of different traits assessed by different interview questions was .15 (heterotrait-heteromethod correlation; discriminant validity). Hence, the average convergent validity coefficient was descriptively higher than the average discriminant validity coefficient. This result speaks in favor of the internal construct-related validity of the present interview when compared with results from previous studies: Previous interview studies found convergent validity coefficients between different interview questions assessing the same constructs to be relatively small, .09 and .05 (both in Huffcutt, Weekley, Wiesner, Degroot & Jones, 2001b), .19 (Van Iddekinge, Raymark, Eidson Jr. & Attenweiler, 2004), and .26 (Klehe, König, Richter, Kleinmann & Melchers, 2008), and to be descriptively smaller than discriminant validity coefficients, .17 and .09 (both in Huffcutt, Weekley, et al., 2001b), 32. (Van Iddekinge et al., 2004), and .41 (Klehe et al., 2008).

As supplementary analysis, we further investigated construct-related validity by examining how personality-based interview ratings correspond to contextualized personality self-reports. Correlational MTMM analyses showed that the average correlation for the same traits assessed by different methods (i.e., by the personality-based interview and the personality self-report) was .34 (monotrait-heteromethod correlation; convergent validity) and the average correlation for the same methods used to assess different traits was .26 (heterotrait-monomethod correlation; discriminant validity). Hence, the average convergent validity coefficient was descriptively higher than the average discriminant validity coefficient.^2^ This speaks for the external construct-related validity of the present interview and stands in contrast to results from previous interview studies that used ratings of individual interview dimensions: Previous interview studies found convergent validity coefficients between individual interview dimensions and questionnaire-based measures assessing the same constructs to be small, .12 (applicant condition in Van Iddekinge et al., 2005) and .21 (Allen et al., 2004), and to be descriptively smaller than discriminant validity coefficients, .50 (applicant condition in Van Iddekinge et al., 2005) and .70 (Allen et al., 2004).

### Predicting OCB: Criterion-related validity of interview ratings

Hypothesis 2 posited that interview ratings of Conscientiousness would predict OCB-compliance over and above the other Big Five traits assessed in the interview. As can be seen in Table 2, Conscientiousness explained a significant proportion of variance in OCB-compliance beyond the other Big Five personality traits, Δ*R*^*2*^ = .04, *F*(1,194) = 9.13, *p* = .003. When including all Big Five traits as predictors of interviewees’ OCB-compliance, only Conscientiousness was significant, *β* = .22, *t*(199) = 3.02, *p* = .003 (see Table 2). In addition, we conducted relative weights analyses using the *relaimpo* package for the R environment (Grömping, 2006) to determine the relative contribution of each Big Five trait towards explaining variance in OCB-compliance. Relative weights analysis is especially useful when multiple predictors are intercorrelated (Johnson, 2000), which is usually the case with interview dimension ratings. Results yielded that 66.8% of the variance that interview ratings of personality traits explained in OCB-compliance is attributable to Conscientiousness, whereas the other traits explained between 0.8 and 22.0% of the variance. As such, Hypothesis 2 was supported.

**Table 2:** Relative weights analyses of OCB-compliance, OCB-helping, and OCB-initiative regressed on interview ratings of the Big Five personality traits

Variables	*B*	*SE B*	*β*	*RW*	*%RW*	*R*^*2*^	Δ*R*^*2*^
OCB-compliance							
Step 1						.024	
Extraversion	− 0.02	0.14	− .01	.001	2.2		
Agreeableness	0.26	0.14	.14	.017	71.6		
Emotional Stability	0.13	0.15	.07	.005	22.2		
Intellect/Openness	− 0.08	0.13	− .05	.001	4.0		
Step 2						.068^*^	.044^**^
Extraversion	− 0.03	0.14	− .02	.001	0.8		
Agreeableness	0.23	0.14	.13	.015	22.0		
Conscientiousness	0.46	0.15	.22^**^	.046	66.8		
Emotional Stability	0.08	0.15	.04	.004	5.4		
Intellect/Openness	− 0.16	0.13	− .10	.003	5.0		
OCB-helping							
Step 1						.068^**^	
Extraversion	− 0.02	0.13	− .01	.003	4.4		
Conscientiousness	0.31	0.15	.15^*^	.030	43.9		
Emotional Stability	0.16	0.14	.08	.012	17.8		
Intellect/Openness	0.21	0.13	.13	.023	34.0		
Step 2						.092^**^	.024^*^
Extraversion	− 0.07	0.13	− .04	.003	2.8		
Agreeableness	0.31	0.14	.17^*^	.035	38.0		
Conscientiousness	0.29	0.15	.14	.027	29.3		
Emotional Stability	0.12	0.14	.06	.010	10.5		
Intellect/Openness	0.15	0.13	.09	.018	19.4		
OCB-initiative							
Step 1						.073^**^	
Extraversion	0.10	0.13	.06	.012	16.4		
Agreeableness	0.31	0.13	.18^*^	.038	51.9		
Conscientiousness	0.08	0.14	.04	.005	6.9		
Emotional Stability	0.19	0.14	.10	.018	24.8		
Step 2						.113^**^	.040^**^
Extraversion	0.00	0.13	.00	.008	7.1		
Agreeableness	0.23	0.13	.13	.029	25.7		
Conscientiousness	0.00	0.14	.00	.003	3.0		
Emotional Stability	0.16	0.14	.08	.014	12.8		
Intellect/Openness	0.37	0.12	.23^**^	.058	51.4		

*Note*: *N* = 200

*RW* relative weights of predictors summing up to *R*^2^, *%RW* percentages of relative weights

^*^p < .05; ^**^p < .01, two-tailed

Hypothesis 3 stated that interview ratings of Agreeableness would predict OCB-helping over and above the other Big Five traits assessed in the interview. Results showed that Agreeableness explained a significant proportion of variance in OCB-helping beyond the other Big Five personality traits, Δ*R*^*2*^ = .02, *F*(1,194) = 5.20, *p* = .024 (see Table 2). When including all Big Five traits as predictors of interviewees’ OCB-helping, only Agreeableness was a significant predictor, *β* = .17, *t*(199) = 2.28, *p* = .024. Relative weights analysis demonstrated that 38.0% of the variance that personality traits explained in OCB-helping is attributable to Agreeableness, whereas the other traits explained between 2.8 and 29.3% of the variance. Based on these results, Hypothesis 3 was supported.

Hypothesis 4 posited that interview ratings of Intellect/Openness would predict OCB-initiative over and above the other Big Five traits assessed in the interview. Table 2 shows that Intellect/Openness explained a significant proportion of variance in OCB-initiative beyond the other Big Five personality traits, Δ*R*^*2*^ = .04, *F*(1,194) = 8.73, *p* = .004. When including all Big Five traits as predictors of interviewees’ OCB-initiative, only Intellect/Openness was significant, *β* = .23, *t*(199) = 2.96, *p* = .004. Relative weights analysis further showed that 51.4% of the variance that personality traits explained in OCB-initiative is attributable to Intellect/Openness, whereas the other traits explained between 3.0 and 25.7% of the variance. Hence, Hypothesis 4 was supported.

Hypotheses 5a to 5c predicted that interview ratings of Conscientiousness, Agreeableness, and Intellect/Openness would explain a significant proportion of variance in OCB-compliance, OCB-helping, and OCB-initiative over and above verbal cognitive ability and the contextualized self-reports of the respective personality traits. In support of Hypotheses 5a to 5c, hierarchical regression analyses revealed that interview ratings of Conscientiousness explained a significant proportion of variance in OCB-compliance beyond interviewees’ verbal cognitive ability and contextualized self-report of Conscientiousness, Δ*R*^*2*^ = .031, *F*(1, 196) = 6.55, *p* = .011. Interview ratings of Agreeableness explained a significant proportion of variance in OCB-helping beyond interviewees’ verbal cognitive ability and contextualized self-report of Agreeableness, Δ*R*^*2*^ = .025, *F*(1, 196) = 5.25, *p* = .023. Finally, interview ratings of Intellect/Openness explained a significant proportion of variance in OCB-initiative beyond interviewees’ verbal cognitive ability and contextualized self-report of Intellect/Openness, Δ*R*^*2*^ = .047, *F*(1, 196) = 10.31, *p* = .002. In each case, personality self-reports became non-significant when personality-based interview ratings as predictors of OCB were included. Results are presented in Table 3. Conversely, personality self-reports did not explain a significant proportion of variance in OCB beyond personality-based interview ratings (when entering personality-based interview ratings first and personality self-reports second into the regression equation). Based on these findings, Hypotheses 5a to 5c concerning the interviews’ incremental validity were supported.

**Table 3:** Relative weights analyses of OCB-compliance, OCB-helping, and OCB-initiative regressed on verbal cognitive ability and on self-reports and interview ratings of Conscientiousness, Agreeableness, and Intellect/Openness

Variables	*B*	*SE B*	*β*	*RW*	*%RW*	*R*^*2*^	Δ*R*^*2*^
OCB-compliance							
Step 1						.036^*^	
Verbal cognitive ability	0.00	0.01	.04	.001	3.0		
Self-report of Conscientiousness	0.45	0.17	.19^**^	.035	97.0		
Step 2						.067^**^	.031^*^
Verbal cognitive ability	0.01	0.01	.04	.001	1.9		
Self-report of Conscientiousness	0.32	0.18	.14	.025	37.8		
Interview rating of Conscientiousness	0.39	0.15	.19^*^	.040	60.2		
OCB-helping							
Step 1						.044^*^	
Verbal cognitive ability	0.01	0.01	.05	.004	9.3		
Self-report of Agreeableness	0.45	0.16	.20^**^	.040	90.7		
Step 2						.069^**^	.025^*^
Verbal cognitive ability	0.01	0.01	.04	.003	4.6		
Self-report of Agreeableness	0.29	0.18	.12	.027	38.7		
Interview rating of Agreeableness	0.31	0.14	.18^*^	.039	56.7		
OCB-initiative							
Step 1						.062^**^	
Verbal cognitive ability	0.02	0.01	.12	.023	37.2		
Self-report of Intellect/Openness	0.37	0.15	.18^*^	.039	62.8		
Step 2						.109^***^	.047^**^
Verbal cognitive ability	0.01	0.01	.10	.018	16.9		
Self-report of Intellect/Openness	0.19	0.15	.10	.026	23.6		
Interview rating of Intellect/Openness	0.38	0.12	.23^**^	.065	59.5		

*Note*: *N* = 200

*RW* relative weights of predictors summing up to *R*^2^, *%RW* percentages of relative weights

^*^p < .05; ^**^p < .01, two-tailed

In addition, to determine the impact of interview length, we explored the interview’s criterion-related and incremental validity using shortened versions of the personality-based interview. Specifically, we tested Hypotheses 2 to 5 measuring each personality traits with (a) one interview question per personality trait (i.e., the one with the highest factor loading) and (b) two interview questions per personality trait (i.e., ratings averaged across the two interview questions with the highest factor loadings). In line with classical test theory (e.g., Gulliksen, 1950), results showed that the interview’s criterion-related and incremental validity increases with the number of interview questions being used to measure each personality trait. The magnitude of correlations between personality predictors (i.e., Conscientiousness, Agreeableness, and Intellect/Openness) and their corresponding types of OCB (i.e., OCB-compliance, OCB-helping, and OCB-initiative) ranged from *r* = .15 (*p* = .032) to *r* = .27 (*p* < .001) when using one interview question per personality trait, from *r* = .19 (*p* = .008) to *r* = .28 (*p* < .001) when using two interview questions per personality trait, and from *r* = .22 (*p* = .002) to *r* = .30 (*p* < .001) when using the original three interview questions per personality trait. In the present sample, Conscientiousness and Agreeableness had to be measured with at least two interview questions to explain a significant proportion of variance in OCB-compliance and OCB-helping over and above personality self-reports and verbal cognitive ability, whereas Intellect/Openness could be assessed with just one interview question to explain a significant proportion of variance in OCB-initiative. Hence, using a shortened version of the personality-based interview will suffice, but it will also come at the cost of slightly reduced criterion-related validity.

Hypotheses 6a to 6c predicted that interview ratings of Conscientiousness, Agreeableness, and Intellect/Openness would mediate the relationships between self-reports of these traits with OCB-compliance, OCB-helping, and OCB-initiative, respectively. We tested these hypotheses applying a bootstrapping method with 20,000 samples (see Preacher & Hayes, 2008) using the *mediation* package for the R environment (Tingley, Yamamoto, Hirose, Keele & Imai, 2014). As can be seen in Table 4, indirect effects (mediation effects) were significant for predicting OCB-compliance, *B* = 0.13 (95% CI = 0.03, 0.28), predicting OCB-helping, *B* = 0.17 (95% CI = 0.01, 0.37), and predicting OCB-initiative, *B* = 0.20 (95% CI = 0.06, 0.37), while direct effects were non-significant. Hence, Hypotheses 6a to 6c were supported.

**Table 4:** Bootstrapping mediation analyses of OCB-compliance, OCB-helping, and OCB-initiative regressed on self-reports and interview ratings of Conscientiousness, Agreeableness, and Intellect/Openness

Model	Estimate	95% CI	*p*
Self-reported Conscientiousness → interview ratings of Conscientiousness → OCB-compliance			
Indirect effect	0.13	[0.03, 0.28]	.009
Direct effect	0.32	[− 0.04, 0.66]	.056
Total effect	0.45	[0.13, 0.77]	.006
Self-reported Agreeableness → interview ratings of Agreeableness → OCB-helping			
Indirect effect	0.17	[0.01, 0.37]	.037
Direct effect	0.30	[− 0.10, 0.70]	.147
Total effect	0.47	[0.11, 0.83]	.011
Self-reported Intellect/Openness → interview ratings of Intellect/Openness → OCB-initiative			
Indirect effect	0.20	[0.06, 0.37]	.003
Direct effect	0.25	[− 0.05, 0.56]	.101
Total effect	0.45	[0.14, 0.78]	.003

*Note*: *N* = 200

*CI* confidence interval

## Discussion

By introducing a personality-based job interview for predicting OCB, our study makes several contributions to the literature. First, our findings demonstrate that interview ratings of specific Big Five personality traits are—to a certain extent—predictive of different types of OCB. This helps to better understand how to select employees for specific citizenship behaviors. For example, results imply that employers may focus on assessing Intellect/Openness in their job interviews if they are specifically looking for employees who are more likely to take initiative in changing and advancing the organization. Second, our findings reveal that all Big Five personality traits can be validly measured using structured interview questions, which goes beyond previous research on assessing personality constructs in the job interview (Van Iddekinge et al., 2005). Third, results show that ratings from a personality-based interview explain some variance in OCB over and above a verbal cognitive ability test and a contextualized personality self-report measure. This suggests that personality-based interviews might have potential to outweigh parts of their costs when compared to other measures (see also Barrick et al., 2000; Macan, 2009; Van Iddekinge et al., 2005). Fourth, findings showed that interview ratings mediated the relationship between self-reported personality traits and corresponding forms of OCB. This implies that personality traits can manifest in employees’ answers to interview questions and that the situation-specific thoughts, feelings, and behaviors that interviewees report in the interview provide some valid information as to whether they are likely to engage in different forms of OCB.

### Theoretical implications

This study adds insights to three different topic areas: dispositional predictors of OCB, personality assessment in selection settings, and the design of structured job interviews. Contributing to research on dispositional predictors of OCB, our findings highlight the benefits of making specific predictions about which personality trait best predicts which type of OCB. Previous research connecting the Big Five personality traits with OCB has often been limited to investigating the role of Conscientiousness and Agreeableness, thereby excluding the predictive role that other traits, like Intellect/Openness, can have (e.g., Ilies et al., 2009; Morgeson, Reider & Campion, 2005; Organ & Ryan, 1995; Venkataramani & Dalal, 2007; Wang & Bowling, 2016). In contrast, the present study included all Big Five personality traits in the analyses, compared two different methods for assessing these personality traits (i.e., interview ratings and self-reports), assessed three specific types of OCB via a third source (i.e., supervisor ratings), and specifically examines the relationship of personality traits and OCB in a simulated selection context. A relatively stable finding, regardless of whether personality traits were assessed with an interview or with a self-report, is that Conscientiousness is most central to predicting OCB-compliance, Agreeableness is most relevant to predicting OCB-helping, and Intellect/Openness is the most important Big Five trait for predicting OCB-initiative.

Contributing to research on personality assessment in selection settings, our results imply that personality-based interview ratings capture criterion-relevant information that a contextualized personality self-report and a cognitive ability measure focusing on verbal reasoning do not capture. In the present study, personality-based interview ratings correlated significantly with self-reports of the same personality constructs and, at the same time, explained variance in OCB beyond self-reports of the same personality constructs and verbal cognitive ability. Hence, personality-based interview ratings share some variance with personality self-reports and also capture additional information on applicants’ personality which helps to predict OCB.

These findings match theoretical underpinnings from the trait-identity-reputation model (McAbee & Connelly, 2016) that considers how the source of information (e.g., the individual, co-workers of the individual, or any other raters) can affect the accuracy and validity of personality judgments. The model distinguishes between variance in personality judgments that is uniquely attributable to self-perceptions of an individuals’ personality (i.e., ‘identity’), others’ perceptions of an individual’s personality (i.e., ‘reputation’), and the actual underlying personality trait defined as the consensus of these different perspectives (McAbee & Connelly, 2016). Within this framework, personality-based interview ratings might be regarded as a special form of others’ perceptions of an applicant’s personality: Interviewers, who have zero acquaintance with the applicant (i.e., strangers), rate applicants’ personality based on applicants’ self-descriptions of their feelings, thoughts, and behaviors in different situations. Thus, interview ratings contain information on how applicants see themselves and on how interviewers perceive and evaluate applicant’s self-views. Therefore, interview ratings could potentially be better indicators of the underlying personality traits (that are defined as consensus from different perspectives) than mere self-reports.

Contributing to job interview research, our findings demonstrate that structured interview questions can be developed to assess established personality constructs and that assessing such well-defined constructs facilitates the construct-related validity of structured interviews (see Hamdani et al., 2014). Selection researchers have repeatedly stated that one of the major challenges in interview research remains to provide construct-related validity evidence (see Hamdani et al., 2014; Klehe et al., 2008; Macan, 2009; Ployhart, 2006; Raymark & Van Iddekinge, 2013; Van Iddekinge et al., 2004). As proposed in a theoretical model by Hamdani et al. (2014), this problem can be addressed by first identifying the criteria one would like to predict and by then theoretically matching these criteria with established, carefully defined, and conceptually distinct predictor constructs that are to be assessed in the interview. The present study followed this approach and presents promising results. Specifically, we found that a factor model specifying only the originally intended interview dimensions as latent factors actually fits the data of an interview administered in a (simulated) selection context. Thus, our findings suggest that choosing well-defined psychological constructs, such as the Big Five personality traits, as interview dimensions is a fruitful approach for developing construct-valid interviews.

### Implications for practice

Given the beneficial effects of OCB on organizational effectiveness (e.g., Podsakoff et al., 2009), our study implies that organizations could benefit from using personality-based job interviews to identify organizational citizens. The most relevant implication is that selection practitioners can use the structured interview questions developed in this study as a blueprint to develop interview questions for selecting employees with a propensity to engage in OCB-compliance, OCB-helping, and OCB-initiative. This will require adapting the interview questions from this study to the respective job. A feasible approach might be to use information gathered from a job analysis to adapt (a) the organizational context described in interview questions and (b) the behavioral anchors so that they refer to behaviors fitting with the demands of the job.

In practice, OCB is not the only criterion on which selection decision would or should be based (see also Organ et al., 2011). Hence, it might be of interest to selection practitioners to use personality-based interview questions for predicting OCB *in addition* to traditional skill-based interview questions for predicting task performance. Specifically, we recommend adding the three interview questions for measuring each OCB-predictor trait (i.e., Conscientiousness, Agreeableness, and Intellect/Openness) if possible. This is because our additional analyses revealed that measuring OCB-predictor traits with fewer interview questions limits the criterion-related validity of the personality-based interview. Using three interview questions per OCB-predictor (resulting in nine interview questions in total) will take at minimum about 18 min of interviewing in addition to the time required for a skill-based interview.

Going beyond the prediction of OCB, there has been an extensive debate about how to assess personality in selection contexts (e.g., Morgeson et al., 2007; Ones et al., 2007). Although the job interview has explicitly been proposed as an alternative personality measure (Barrick et al., 2000; Levashina et al., 2014; Raymark & Van Iddekinge, 2013; Van Iddekinge et al., 2005), this study is first to provide both construct-related and initial criterion-related evidence for a structured interview designed to assess the Big Five personality traits. Our findings imply that practitioners can use interview questions to validly assess personality in selection settings (with the purpose of predicting different kinds of criteria such as person-group fit, job satisfaction, training success, etc.), which seems especially relevant given that job interviews are an integral part to standard selection procedures (Di Milia, 2004; König et al., 2010; Levashina et al., 2014).

### Limitations

Of course, this study is not without limitations. First and foremost, interviewees were not actual applicants. Instead, they were interviewed for a fictitious job in a simulated selection setting. Therefore, interviewees might have been less motivated to perform well in the interview as compared to their performance during an actual job interview. With this limitation in mind, we still chose to conduct the study in a simulated setting so that we could have control over the information on which interviewers base their personality ratings (see also the study design in Barrick et al., 2000; Klehe et al., 2008; Swider et al., 2016; Van Iddekinge et al., 2005). Although interviewees participated voluntarily in this study to receive valid performance feedback that would help them with future job applications, their motivation to perform well would presumably still be high. In fact, our data indicated that interviewees mostly behaved as if they were in an actual selection process and that the interview was criterion valid, which might lower concerns about the generalizability of results.

A second limitation is the heterogeneity of the sample in the present study. Interviewees were from different organizations and held a variety of jobs. Hence, OCB ratings might not be fully comparable across different interviewees, given that OCBs can depend on the organizational context and specific work environment (e.g., on job autonomy, job meaning, and on the quality of social exchange relationships at the workplace; Kamdar & Van Dyne, 2007; Liguori, McLarty & Muldoon, 2013). At the same time, the heterogeneity of the sample might speak for the generalizability of the present findings across different kinds of jobs and work environments.

Third and relatedly, the focal relationships between interview ratings of personality traits and supervisor ratings of OCBs were only moderate ranging from *r* = .22 to *r* = .30. These effect sizes can be categorized as small to medium (Bosco, Aguinis, Singh, Field & Pierce, 2015; Cohen, 1992) and are slightly lower than uncorrected estimates reported in the latest meta-analyses on the validity of medium to highly structured job interviews ranging from $\overline{r}$ = .25 to $\overline{r}$ = .36 (Huffcutt et al., 2014; Thorsteinson, 2018). One explanation may be the heterogeneity of the present sample (i.e., interviewees held different jobs and interview questions were not tailored to meet unique demands of these specific jobs). An alternative explanation might be that structured interviews could work slightly better for predicting task performance (which has been a focal criterion in previous interview studies; Thorsteinson, 2018) as compared to predicting OCB, but more research is needed to test this assumption.

Finally, intercorrelations between interview ratings and personality self-reports found in the present study can be classified as modest to moderate (ranging from *r* = .18 to *r* = .45). An explanation for this might be that structured interviews and self-reports differ substantially with regard to several method factors (Heimann and Ingold, 2017; Lievens & Sackett, 2017). For example, they differ with regard to (1) the person providing the rating (trained interviewer versus untrained interviewee/applicant), (2) their stimulus formats (verbal interview questions versus written questionnaire items), and (3) their level of contextualization (descriptions of specific situations versus more generic items). This might limit expectations about the convergence of structured interviews and self-reports as each uses a different measurement approach. In line with, and similar to, the present study, previous interview research found modest relationships between traditional job interviews and personality self-reports (Roth et al., 2005; Salgado & Moscoso, 2002).

### Directions for future research

To increase our understanding of how to best predict OCB in job interviews, future research may directly compare interview questions assessing personality traits with interview questions designed for assessing OCBs. So far, previous studies assessing OCBs with behavior description interview questions were only partly successful (Allen et al., 2004; Latham & Skarlicki, 1995). An advantage of personality-based interview questions over OCB-based interview questions might be that personality traits are supposed to be more stable predictors across various situations as opposed to OCBs (Cohen, Ben-Tura & Vashdi, 2012; Fassina, Jones & Uggerslev, 2008). In addition, it might be more common and legally accepted to assess personality traits in selection settings (e.g., Dilchert, Ones & Krueger, 2019), whereas the conditions are less clear when it comes to explicitly assessing OCBs in a selection context (for an overview see Organ et al., 2011). However, more research is needed to understand whether and how the criterion-related validity of personality-based and OCB-based interview questions differs.

Relatedly, research is needed to explore how interview questions that were originally designed to predict OCB relate to task performance. This refers to the question of whether there is a trade-off between OCB and task performance. It is possible that employees who dedicate a substantial amount of their resources to OCBs will have less capacity for demonstrating high levels of task performance or will even engage in counterproductive work behavior to compensate for their efforts (e.g., Bolino, Klotz, Turnley & Harvey, 2013). What speaks against a trade-off between predicting OCB and predicting task performance by the same interview questions is that, conceptually, OCBs are thought to create an environment that enables task performance (Organ, 1997), and empirically, previous research has found strong relationships between employees’ OCB and task performance (Hoffman et al., 2007). Hence, more research is needed to investigate whether selecting organizational citizens would also result in the selection of high performers.

## Conclusion

OCB goes beyond the completion of individual work tasks and can determine how well employees function together and form a successful organization. Yet, in the context of personnel selection, our means for identifying those employees who are willing to go the extra mile have been limited. In this context, the present study demonstrates that a structured job interview assessing the Big Five personality traits is a suitable measure for predicting different types of OCB. Thus, we encourage practitioners to use carefully developed and job-related interview questions for selecting organizational citizens, and we propose to selection researchers to further expand the criterion domain when validating new selection instruments—by considering OCB, as well as other relevant criterion constructs (i.e., employee well-being and commitment) in addition to task performance.

## Appendix 1. Example behavior description interview question for extraversion

“Sometimes you meet a lot of new people. Think of a situation where you participated in a oneor two-day training or workshop and where you did not know the other participants, but some of them seemed very friendly. Please describe exactly how you perceived this situation and what you did in this situation to interact with the other participants”

Dimension: *Extraversion*

5 Feels enthusiastic about the opportunity to make new contacts – actively tries to get to know as many other participants as possible – makes use of every opportunity to make new contacts – surrounds himself or herself with different people
3 Perceives the situation as rather interesting – is generally open towards meeting other participants – needs a reason or a push from others to make new contacts – surrounds himself or herself with people they know if possible
1 Feels rather uncomfortable in this situation – is reserved towards other participants – does not try to make new contacts – avoids interactions with others – stays alone or around people he or she already knows

## Appendix 2. Example behavior description interview question for Agreeableness

“Sometimes we notice that someone else makes a mistake. Think of a situation when a co-worker made a mistake and you pointed the mistake out to them. Please describe exactly how you perceived this situation and what you did in this situation when you spoke to your co-worker.”

Dimension: *Agreeablenss*

5 Is very careful in pointing out the mistake – thinks that it is a priority to be considerate regarding the co-workers’ emotions – avoids accusations – points out that everyone makes mistakes
3 Politely and directly points out the mistake – thinks it is important to be considerate regarding the co-workers’ emotions – does not point out that everyone makes mistakes sometimes
1 Directly points out the mistake – does not think that it is important to be considerate of the co-workers’ emotions – does not see the emotional side to this situation – makes accusations or behaves in an insulting manner

## Appendix 3. Example behavior description interview question for Conscientiousness

“Everyone has an individual work style and standards. Please think of a situation when you were working on the last steps before handing in a piece of work (e.g., a report or any kind of written assignment). Please describe exactly how you perceived this situation and what you did in this situation regarding the completion of this piece of work.”

Dimension: *Conscientiousness*

5 Proceeds in a very systematic and structured manner – thinks that it is most important to strive for accomplishment – plans ahead and accounts for buffer time – follows a detailed schedule – double-checks his or her work several times before handing it in
3 Generally proceeds in a systematic and structured manner – thinks that it is important to strive for accomplishment – plans individual work steps – follows a schedule – doublechecks his or her work roughly before handing it in
1 Proceeds in an unsystematic and unstructured manner – does not think that it is important to strive for accomplishment – does not plan individual work steps – does not follow a schedule – does not double-check his or her work before handing it in

## Appendix 4. Example behavior description interview question for Emotional Stability

“Sometimes you have to wait longer than you thought would have to. Think of a situation that ended with you not getting the information that you urgently needed – even though you had asked for this piece of information several times. Please describe exactly how you perceived this situation and what you did in this situation regading the missing information.”

Dimension: *Emotional Stability*

5 Hardly feels any inner tension regarding the missing information – does not take it personally – behaves in a polite manner towards the responsible person – searches for other ways to get the missing piece of information in a calm and constructive way
3 Feels frustrated regarding the missing information – feels treated unfairly – still behaves in a polite manner towards the responsible person – tries to constructively search for other ways to get the missing piece of information
1 Feels angry regarding the missing information – feels personally attacked or thinks of himself or herself as a victim – reacts very emotionally and in an uncontrolled manner towards the responsible person – is not able to constructively search for solutions

## Appendix 5. Example behavior description interview question for Intellect/Openness

“Some subjects may seem especially interesting. Please think of a situation when you had the opportunity to look into a new subject more deeply than was necessary for completing a task. Please describe exactly how you perceived this situation and what you did in this situation regarding the opportunity to look into the new subject.”

Dimension: *Intellect/Openness*

5 Feels enthusiastic about looking deeply into any new subject – is completely open to new ideas – can inspire and motivate himself or herself to approach new subjects
3 Feels that looking more deeply into certain subjects may be interesting – is willing to open up to new ideas to a certain extent – can generally be inspired and motivated to take a step towards new subjects
1 Hardly feels the need to deeply look into the new subject – is not willing to open up to new ideas – can hardly be inspired and motivated to take a step towards new subjects

## Footnotes

1 Please note that Van Iddekinge et al. (2005) did not find support for a factor model reflecting their originally intended interview dimensions because the correlation between two of their dimensions exceeded unity. The fit mentioned here refers to their modified model that specifies only two out of the original three interview dimensions.

2 In addition, we conducted an MTMM CFA across personality-based interview ratings and contextualized personality self-reports to estimate the amount of variance in personality ratings that was attributable to trait factors (i.e., the Big Five personality traits) versus method factors (i.e., the structured interview and contextualized self-report). To calculate this model, we created parcels from the personality self-report. More precisely, we randomly assigned the ten items used to measure each trait to one of three parcels per trait (Little, Cunningham, Shahar & Widaman, 2002). In summary, fit indices implied modest fit, *χ*^2^/*df* = 1.58, *p* < .001, CFI = .89, TLI = .87, RMSEA = .05, and SRMR = .08. Results indicated that average factor loadings were .49 for trait factors (with average loadings ranging from .27 for Emotional Stability to .59 for Extraversion) and .38 for method factors (with average loadings being .25 for interview ratings and .67 for self-reports). Factor loadings imply that 23.6% of the variance in personality ratings was explained by trait factors and 14.2% of the variance in personality ratings was explained by method factors.
